# An object simulation model for modeling hypothetical disease epidemics – EpiFlex

**DOI:** 10.1186/1742-4682-3-32

**Published:** 2006-08-23

**Authors:** Brian Hanley

**Affiliations:** 1 BW Education and Forensics, 2710 Thomes Avenue, Cheyenne, Wyoming 82001, USA

## Abstract

**Background:**

EpiFlex is a flexible, easy to use computer model for a single computer, intended to be operated by one user who need not be an expert. Its purpose is to study in-silico the epidemic behavior of a wide variety of diseases, both known and theoretical, by simulating their spread at the level of individuals contracting and infecting others. To understand the system fully, this paper must be read together in conjunction with study of the software and its results. EpiFlex is evaluated using results from modeling influenza A epidemics and comparing them with a variety of field data sources and other types of modeling.

EpiFlex is an object-oriented Monte Carlo system, allocating entities to correspond to individuals, disease vectors, diseases, and the locations that hosts may inhabit. EpiFlex defines eight different contact types available for a disease. Contacts occur inside locations within the model. Populations are composed of demographic groups, each of which has a cycle of movement between locations. Within locations, superspreading is defined by skewing of contact distributions.

**Results:**

EpiFlex indicates three phenomena of interest for public health: (1) R_0 _is variable, and the smaller the population, the larger the infected fraction within that population will be; (2) significant compression/synchronization between cities by a factor of roughly 2 occurs between the early incubation phase of a multi-city epidemic and the major manifestation phase; (3) if better true morbidity data were available, more asymptomatic hosts would be seen to spread disease than we currently believe is the case for influenza. These results suggest that field research to study such phenomena, while expensive, should be worthwhile.

**Conclusion:**

Since EpiFlex shows all stages of disease progression, detailed insight into the progress of epidemics is possible. EpiFlex shows the characteristic multimodality and apparently random variation characteristic of real world data, but does so as an emergent property of a carefully constructed model of disease dynamics and is not simply a stochastic system. EpiFlex can provide a better understanding of infectious diseases and strategies for response.

## Background

This paper is intended to be read along with a working copy of the EpiFlex software, (see Additional file 1). It describes the context of the work, an overview of the system design, a discussion of certain primary mechanisms, and examples of observations made using the system. EpiFlex was designed to be as easy to use as possible and is intended to be usable by non-experts, though experts would be expected to gain greater insight and understanding from it. Much of what it presents requires significant study and preliminary training before it can be used effectively and understood. EpiFlex can be an effective aid to teaching. Availability of source code can be discussed on a case by case basis with the author. Such collaborators are desired. Epiflex is written in C++ for Windows platform at this time.

### Context of this work

This work is related to several threads within modeling and simulation. Giro et al. [1] proposed detailed discrete modeling of ecosystems; Ginovart et al. [2] developed INDISIM, a discrete simulation of bacterial colonies; Eubank et al. [3] and Barret et al. [4] discuss EpiSims-related work [5]. EpiSims is a Los Alamos project for discrete modeling of epidemics in cities, starting with Portland, Oregon. It was developed in relationship to the TRANSIM model for understanding movements of people in cities. According to press releases, there is similar work at Emory University aimed at developing a model of disease in hypothetical American communities of 2,000 to 48,000 people [6]. Johns Hopkins University has a program that has been funded to accomplish similar goals [7]. A number of authors including Schinazi [8], Aparicio et al. [9] and others [10,11] have explored clustering in the real world and its relevance to the spread of disease, as well as theoretical models. EpiFlex has modeled communities with multiple demographics linked by transport corridors for population sizes up to 3.5 million. This is not the limit; large models can be quite slow to execute, but EpiFlex can be scaled up given enough computing resources. This will happen to some degree as Moore's law provides faster computers with more memory. Additionally, the internal architecture of EpiFlex was designed with parallelization of modules in mind, so it should be fairly straightforward to do modify given resources. However, some of the more interesting results are obtained from lower order population sizes where "small world networks" [11,12] can have interesting impacts, and models can show differences in morbidity linked to population size. Watts has criticized mathematical models as inadequate to show real world variation in epidemics [13].

### Discussion of R_0_

The most commonly used measure in public health, R_0_, is estimated from historical data and derived from SIS/SIR type models (and descendents) for forward projection[14,15] R_0 _is the basic reproductive ratio for how many individuals each infected person is going to infect[16] R_0 _is often used on its own in public health as an indicator of epidemic probability; if R_0 _< 1 then an epidemic is not generally considered possible, for R_0 _> 1, the larger the value, the more likely an epidemic is to occur. R_0 _is a composite value describing the behavior of an infectious agent. Hence, R_0 _can be decomposed classically, for example, as: p d c, where p is probability of infection occurring for a contact, d is duration of infectiousness, and c is number of contacts[17].

However, R_0 _in the classical decomposition above, while it is one of the best tools we have, does not account for age segregation of response, existing immunity in population, network topology of infectious contacts and other factors. These observations were significant in the motivation for developing EpiFlex.

### Design of EpiFlex

The EpiFlex model was designed to create a system that could incorporate as much realism as possible in an epidemic model so as to enable emerging disease events to be simulated. There are limitations, described below in a separate section, but the model is quite effective as it stands. In most cases, the limitations of EpiFlex are shared by other modeling systems.

There are a variety of methods used for mathematical modeling of diseases. The most common of these are the SIR (susceptible, infected, recovered) of Kermack and McKendrick [15], SIS (susceptible, infected, susceptible), SEIR (susceptible, exposed, infected, recovered), and SIRP (susceptible, infected, recovered, partially immune) as developed by Hyman et al. [18] and further developed by Hyman and LaForce[19]. The SIRP model was used as the starting point for development of the object model of EpiFlex. In SIRP, the SIR model is extended to include partial immunity (denoted by P) and the progressive decline of partial immunity to allow influenza to be modeled more accurately. (See Appendix.)

There is a need for experimentation in more realistic discrete modeling, since the lattice type of discrete modeling is understood to skew in favor of propagation, as discussed by Rhodes and Anderson [20] and Haraguchi and Sasaki [21]. Others such as Eames and Keeling [22] and Edmunds et al. [12] have explored the use of networks to model interactions between infectable entities, and Ferguson et al. [23] and others have called for more balance in realism for epidemiology models. Since EpiFlex was completed, Lloyd-Smith et al. [17] have shown the importance of superspreading in disease transmission for the SARS epidemic. EpiFlex is designed to take these issues into account.

There are known weaknesses in SIS-descended models, some of which are discussed by Hyman and LaForce [14]. They suggested that a model dealing with demographics and their subgroups would be useful and described a start toward conceiving such a model, creating a matrix of SIRP flows for each demographic group within a "city" and modeling contacts between these groups. Thus, the possibility of building an entirely discrete model using the object-oriented approach, essentially setting the granularity of the Hyman-LaForce concept at the level of the individual, together with Monte Carlo method, was attractive. The object method of design seemed to be a good fit, since object-oriented programming was invented for discrete simulations [24]. An Object-Oriented (OO) design defines as its primitive elements "black box" subunits that have defined ways of interacting with each other [25].

The OO language concept originally was conceived for the Simula languages [24] for the purpose of verifiable simulation. Enforcement of explicit connections between objects is fundamental to OO design, whereas procedural languages such as FORTRAN and COBOL do not because data areas can be freely accessed by the whole program. OO languages wrap data in methods for accessing the data. If each "black box" (i.e. object) has a set of specified behaviors, without the possibility of invisible, unnoticed interactions between them, then the simulation can potentially be validated by logical proof in addition to testing. (It would take an entire course to introduce OO languages and concepts, and there is not space to do so here. Interested readers are suggested to start with an implementation of Smalltalk. There are excellent free versions downloadable. Smalltalk also has an enthusiastic and quite friendly user community. See: .)

## Models and methods

The design of EpiFlex is described more completely in the appendix. Design proceeded by establishing the definition of a **disease **organism as the cornerstone, then defining practical structures and objects for simulating the movement of a disease through populations. The **disease **object was assigned a set of definitions drawn from literature that would allow a wide spectrum of disease-producing organisms to be specified. The aim was to minimize the number of configuration parameters that require understanding of mathematical models.

The **hosts **that are infected became the second primary object. A **host **lives and works in some **area**, where hosts are members of some **demographic group**, which together determine what of *n *types of **contacts **they might have to spread an infectious **disease**. The **hosts **move about the **area **in which they live between **locations **at which they interact. In EpiFlex, an **area **contains some configured number of **locations**, and **locations **are containers for temporary groups of **hosts**. Since people travel between metro **areas**, the model supports **linkages **between **areas **to move people randomly drawn from a configurable set of demographic groups.

The remainder of this section presents the disease model adopted, an overview of each component, an overview of program flow, and a description of the core methods. This is followed by discussion of results from the EpiFlex software system.

### Disease model

**Figure 1:**
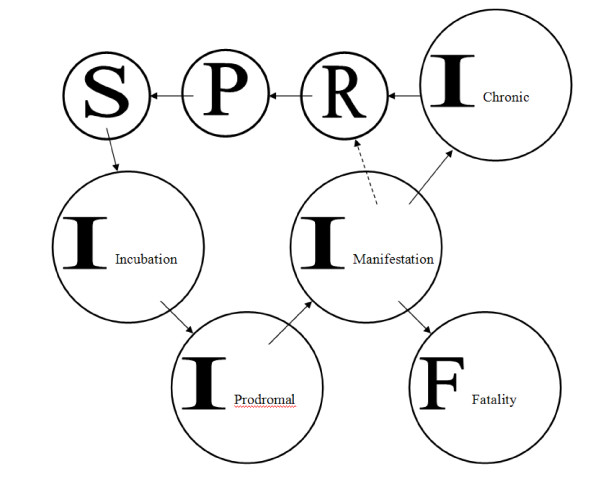
Extended-SIRP disease model of Epiflex. S: susceptible I: Infected R: recovered P: partially immune F: fatality Extended SIRP breaks the infected stage I into 4: I_Incubation_, I_Prodroma_l, I_Manifestation_, I_Chronic_, and adds a fatality terminating stage. .

This model has up to four stages during the infection cycle: the Incubation, Prodromal, Manifestation, and Chronic stages; to this is added a fatality phase. I have named this 'extended-SIRP'. Fig. 1 shows a diagram of this model.

The model of Fig. 1 allows us to track the different phases of the disease process separately, and to define variable infectiousness, symptoms, fatality, recovery and transition to chronic disease at each stage as appropriate. This allows us to model the progress of a disease in an individual more realistically. For diseases that have no identifiable occurrence of a particular stage, this stage can be set to length zero to bypass it entirely.

### Contact types in disease model

The 8 contact types designed into EpiFlex are drawn from literature in an attempt to model spread of infection more accurately. These contact types are: blood contact by needle stick, blood to mucosal contact, sexual intercourse, skin contact, close airborne, casual airborne, surface to hand to mucosa, and food contact. The probability of infection for a contact type is input by the user as estimated from literature or based on hypothetical organism characteristics.

### Monte Carlo inputs to disease model

Durations of disease stages are chosen uniformly at random from a user-specified interval [**R**_**low**_, **R**_**high**_]. Random numbers, denoted by ξ, on [0, 1] are used to seed the determination of the infected disease stage periods (denoted I_Incubation_, I_Prodromal_, I_Manifestation_, I_Chronic_). **R**_**low **_and **R**_**high **_are taken from medical literature and describe a range of days for each stage of an illness. These calculations are simply: **(ξ × (R_**high **_- R_**low**_)) + R_**low **_= D**, where D is days for a particular stage. (This may be extended in the future to include ability to define a graph to determine the flatness of distribution and the normative peak. This will make a significant difference in modeling of diseases such as rabies, which can, under unusual circumstances, have very long incubations.)

One of the following three equations describing immunity decay is chosen; L is the current level of partial immunity, P is the level of partial immunity specified as existing immediately following recovery, Δ is number of days since recovery, D is the duration in days of the partial immunity stage, and C is a constant chosen by the user to describe the shape of the asymptotic curve in choice 3.

1. if (Equation = No Decline) then L = P

2. if (Equation = Linear Decline) then L = P × Δ/D

3. if (Equation = Asymptotic Decline) then L = P (1 - (1 - (Δ/D) ^**C**^)

When L ≤ 0 then L = 0.

Random values on [0, 1] are then used to decide whether an infection occurs during the partial immunity phase **P **shown in the chart above. This decision uses the output of the immunity level algorithm, **L, **which is a number on [0, 1], as is the random value ξ:

**if (ξ > L) then ***infection has occurred*.

### Location contact distributions for infection modeling

EpiFlex uses a dynamic network to model the interactions between hosts at a particular location based on the skew provided and the demographic segments movement cycles. The networks of contacts generated in this version of EpiFlex are not made visible externally; they can only be observed in their effects. (See: Limitations of EpiFlex modeling.) Their algorithms were carefully designed and tested at small scales, observing each element.

A location describes a place, the activities that occur there, and the demographic groups that may be drawn there automatically. A location can have a certain number of cells, which are used to specify N identically behaving locations concurrently. This acts as a location repetition count within an area when the location is defined. The user sets an average number of hosts inhabiting each cell, and a maximum. There is also a cell exchange fraction specifiable to model hosts moving from cell to cell. The algorithm for allocating hosts in cells is semi-random. It randomly puts hosts into cells in the location. If a cell hits the average, then it does another random draw of a cell. If all locations are at maximum, then it overloads cells.

Interactions are within the cell. So a host must be exchanged to another cell in order to be infective. See the appendix for 'Location component', and also with an open model look at how hospitals were defined. Households are modeled at this time using a cell configuration.

### Monte Carlo algorithm

EpiFlex is implemented with a Monte Carlo algorithm such that each host in a location is assigned a certain number of interactions according to the Cauchy distribution parameter setting for that location. This distribution describes a curve with the *y *axis specifying the fraction of the maximum interactions for the location and *x *axis specifying the fractional ordinal within the list of hosts in the location. The distribution can be made nearly flat, or severely skewed with only a few actors providing nearly all contacts, as desired by the user of EpiFlex. Note that the structure of the network formed also depends on what locations are defined, what demographic groups are defined for the population, and how demographic groups are moved between locations. Each location has a maximum number of interactions specified per person, which is used as the base input. Initially, a Gaussian equation was used, but it was discarded in favor of a Cauchy function since this better fits the needs of the skew function and computes faster. The algorithm iterates for each infectious host, and selects other hosts to expose to the infected party in the location, by a Monte Carlo function. This results in a dynamically allocated network of interactions within each location.

#### Exposure cycle

The exposure cycle also makes use of Monte Carlo inputs. Each location has a list of contact types that can take place at a particular location, and a maximum frequency of interactions. This interaction frequency determines how many times contacts that can spread a disease will be made, and the contact specification defines the fractional efficacy of infection by any specific route. Modeling the effect of different types of contacts has been discussed in the literature, e.g. Song et al. [26]. EpiFlex attempts to make a more generalized version.

For each host infection source, target hosts are drawn at random from the location queue. A contact connection is established with the target as long as the contact allocation of that target has not been used up already. Contact connections made to each target are kept track of within the location to prevent over-allocation of contacts to any target. Thus, for each randomly established connection, a value is set on both ends for the maximum number of connections that can be supported. Once the maximum for either end of the link is reached, the algorithm will search for a different connection.

### Cauchy distribution

The location algorithm is described below in more detail. The user specifies the maximum number of connections for a location; the **σ **output from a Cauchy distribution function determines how many connections an individual will have. This allows variations in the degree of skewness for superspreading in a population to be modeled, which has been shown to be of critical importance by Lloyd-Smith et al. [17].

If p = position in queue, q = number of hosts in queue for location:

**X = p/q, **where X denotes the proportional fraction of queue for position.

If K is a constant chosen for the location to express skew distribution, the Cauchy distribution function is:

**σ = K^**2**^/(K^**2 **^+ X^**2**^) **since we want a normal on [0, 1]

If κ is the number of contacts for a particular host and κ_max _is maximum number of contacts for any given host in the location:

κ = κ_**max **_× σ

When hosts move from one location to another within the model, they tend to maintain a rough order of ordinal position. Consequently, when there is a high **σ **for a location, the high connection host in one location tends to be a high connection host in another. This reflects real-world situations, (though not perfectly) and corresponds better than persistently maintaining high connection individuals from location to location, since host behavior changes from place to place.

The Cauchy distribution function is fairly fast in execution. The function can be used to approximate the often radical variations seen in epidemiology studies; as an extreme example, one active super-spreader individual might infect large numbers, when one or even zero is typical [17]. This type of scale-free network interaction has been explored by Chowell and Chavez [27]. The Cauchy function allows networks to be generated dynamically within each type of location in a very flexible manner, such as corresponding to super-spreader dynamics [17]. In addition to the specification of skew within a location, the network of contacts is also defined by (a) what locations are present and (b) the movement cycles defined for each demographic group within the model.

### Processing time is primarily the series sum of infection modeling events

Processing time increases with population. This slowing is an expected characteristic of an object modeling system and is the price paid for the discrete detail of the EpiFlex model. The primary source of this increase in processing time is the sum of series of possible infectious events that are modeled for each iteration. It therefore scales as a series sum not as a log, based on the contagiousness of the disease and the number of potential hosts in a location with an infected host. This is minimized by only processing infectious host contacts. The increase stems from the characteristics of networks in which each node has *n *connections to other nodes. When iteration is done for a location containing infectable hosts, it is the number of infected hosts that creates an element of the series. The infected hosts are put into a list, and each one interacts randomly with other hosts (including other infected ones) in the location. Thus, considered as a network with *m *nodes, each of the *m *nodes is a host. A temporary connection to another host is made to *n *other nodes where *n *<*m*, and *n<k*. The value of *k *is determined by a randomized input that returns the number of contacts of this infected host in this location. Consequently, the series consists of all the temporary connections made for contact modeling for each cycle.

### Limitations of EpiFlex modeling

In the interest of completeness, the limitations of the EpiFlex model are described here. The plan is to address these elements for implementation in future versions.

#### One disease at a time

Only one infectious disease can be occurring at a time. Thus, competitive inhibition [28] and synergistic effects will not be seen.

#### One type of host

Only one kind of host can exist. Multiple hosts are needed to model zoonoses optimally. EpiFlex can imitate zoonoses to some extent by defining a 'vector' within the model in various ways. (See Appendix, 'Initiating Disease Vector Component')

#### Hosts do not reproduce

Hosts do not reproduce within a model. Removal and addition rates are defined for the population as a whole, and the basis is US Census data. To meet the specifications for removal and addition within the model, hosts are removed from randomly chosen locations, and similarly added to randomly chosen locations. Demographic group is also randomly assigned. For long-term modeling, and modeling of alternative short-lived hosts, a reproduction cycle is desirable. However, EpiFlex is a practical way of modeling periods of a few years.

#### No explicit definition of age distribution

There is no explicit definition of an age distribution for the host population, which can be quite significant [29]. To a degree, age is taken into account through the demographic segmentation of populations. A demographic can be defined with a fraction or multiple of baseline susceptibility. However, hosts do not age, nor do they move from one demographic to another as they age.

#### Previous exposure profile for hosts and complex antigen specification are not provided

No provision is made to define a previous exposure profile for hosts [30,31]. In real populations previous exposures can have significant effects on the spread of a disease and dramatic effects on mortality where infection does occur [32]. Proper implementation of previous exposure profiles is intertwined with age definition.

#### Disease mutation not modeled – rolled into immunity decay

There is no implementation of mutation rate for diseases. Mutation rates vary considerably by type, particularly for viruses [33]. Decay of immunity is modeled, and immunity decay can act as a fair surrogate for antigenic change.

#### Pass-through events must be defined as part of surface contacts

For efficiency, EpiFlex eliminates pass-through infection events from being modeled: for example, an infected A shakes the hand of a non-infected B, who then shakes the hand of another non-infected C, but B washes hands and does not become infected while C does. Therefore, the model definition must account for this through "Surface to hand to mucosa" contacts, where a person can also be a surface.

#### Network of contacts not easily available within locations

The model does not at this time record the contact network that is dynamically created except in the log file at this time. Those that are logged are only potential infectious contacts. To get at that data requires looking at the log file and writing an extract program. Making the network visible is an item for the future.

#### Seasonal damping cycle not provided

Currently, EpiFlex has no way of accounting for seasonal damping. Similarity of results is due to the settings of the rate at which immunity declines. Addition of a seasonal damping function would be expected to cause EpiFlex results to synchronize with a yearly cycle. Seasonal damping would result in loss of interesting epidemic behavior with an overriding function that would virtually be guaranteed to drown out other behaviors.

#### Public health response to epidemics is not optimally modeled

A public health response definition component is present in EpiFlex. Testing of this component, and more thorough review of literature, indicate that the method used is not optimal. Current public health responses are centered on contact tracing, ring vaccination and quarantine [34], with mass vaccination as a backup when it is available. Closures of schools, daycare and travel restrictions are also used. These methods are not modeled in EpiFlex' response component. Their importance has recently been underscored by Lloyd-Smith et al. [17]. As a consequence, results from the current system that defines across-the-board cuts in the probability of infection should be considered in this light. It is not clear whether any other object system can model all current techniques properly.

#### Distribution of disease stage times is flat

Diseases have ranges of times for each stage that can be drawn from literature. Probability of a specific disease stage time period for an infected host being chosen within the range is equal. This is reasonably adequate for most diseases where times are measured in a few days, however, some, such as rabies have a quite unequal distribution, and their very long tail makes a difference in modeling.

## Discussion

The discussion is presented in three parts: (1) a brief set of examples of native EpiFlex displays to develop a better feel for the system; (2) comparisons of EpiFlex results with real world data; (3) a set of examples of observations made using EpiFlex. The purpose of these examples is to serve as a guide to others who may want to experiment and analyze results.

### EpiFlex display data

**Figure 2:**
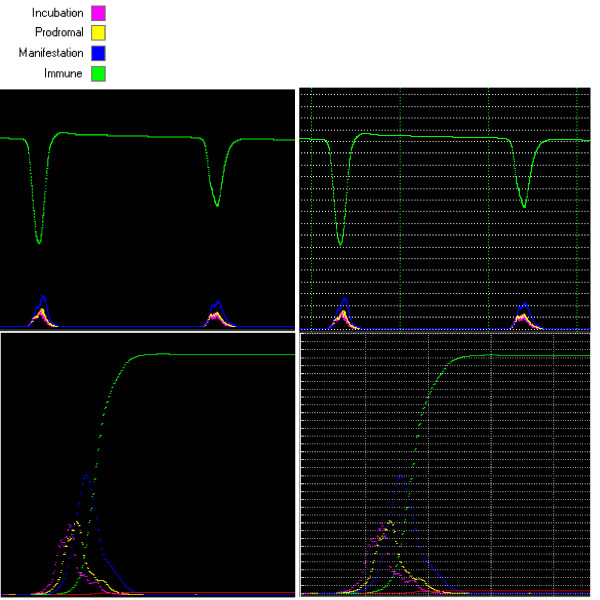
(Clockwise, a, b, c d) – Part of a multi-year simulation display for a city of 350,000 people. (2a., 2b.) Two alternative displays. Vertical scale demarcation is 10,000. Horizontal scale one year per demarcation. Simulation specifies asymptotic immunity decay period of 730 days. Intention is to simulate a virus with mutation leading to major epitope change over a period of 600 to 730 days. A continuous seeding of 3 attempts to infect a college student each day was defined. (2c. 2d.) A simulation of a single influenza epidemic in a 35,000 population. Vertical scale 1,000. Horizontal scale one month per demarcation.

Different views of the epidemic data for simulated influenza in two different populations are shown in Figure 2. Figures 2a and 2b show graphs of the second and third epidemics in the population. These graphs show the kinds of commonly-seen deviations from a smooth curve that occur in real world data [35]. In the EpiFlex model, this is attributed to less synchronization of immunity combined with the formation of small world networks among demographic groups as they move from location to location.

**Figure 3:**
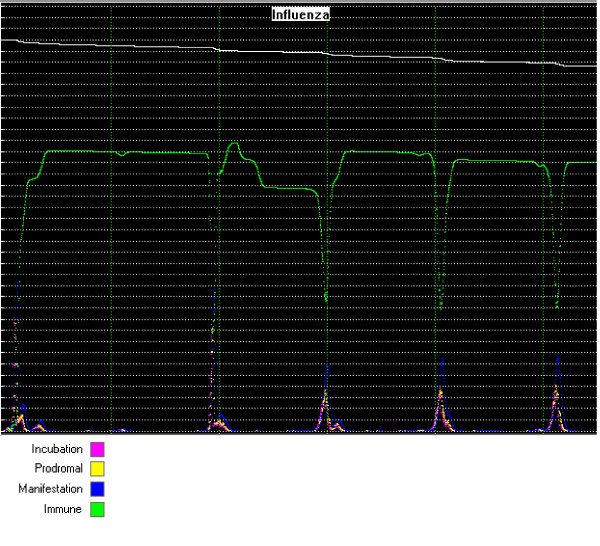
This is the simulation that was imported for comparison. Vertical scale 1000 per demarcation. Horizontal scale one year per demarcation. Upper white line is total population with standard removal rate.

Figures 2c and 2d are alternate views of a simple influenza epidemic occurring within a naïve population (Figure 3).

### Comparisons with real world data and a mathematical model

#### Comparison of EpiFlex with WHO/NREVSS surveillance

**Figure 4:**
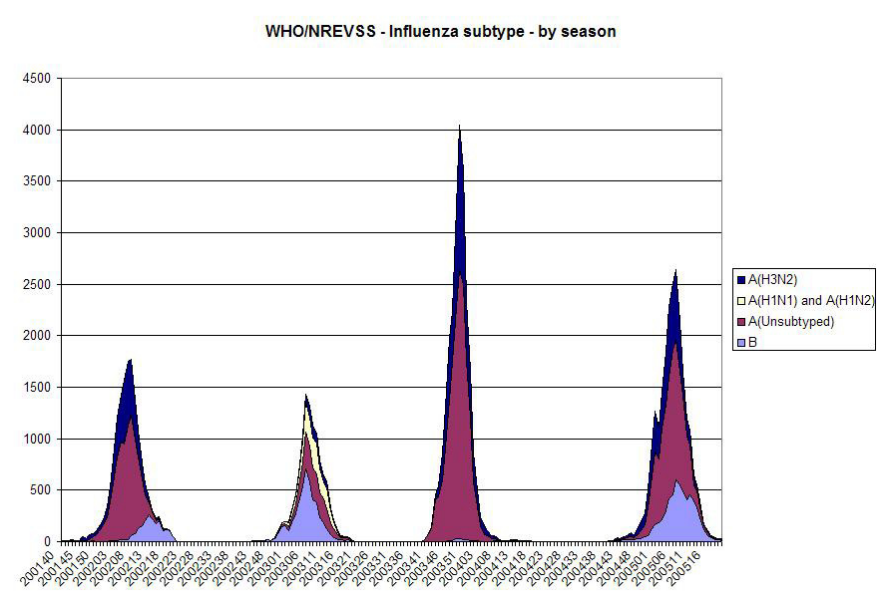


**Figure 5:**
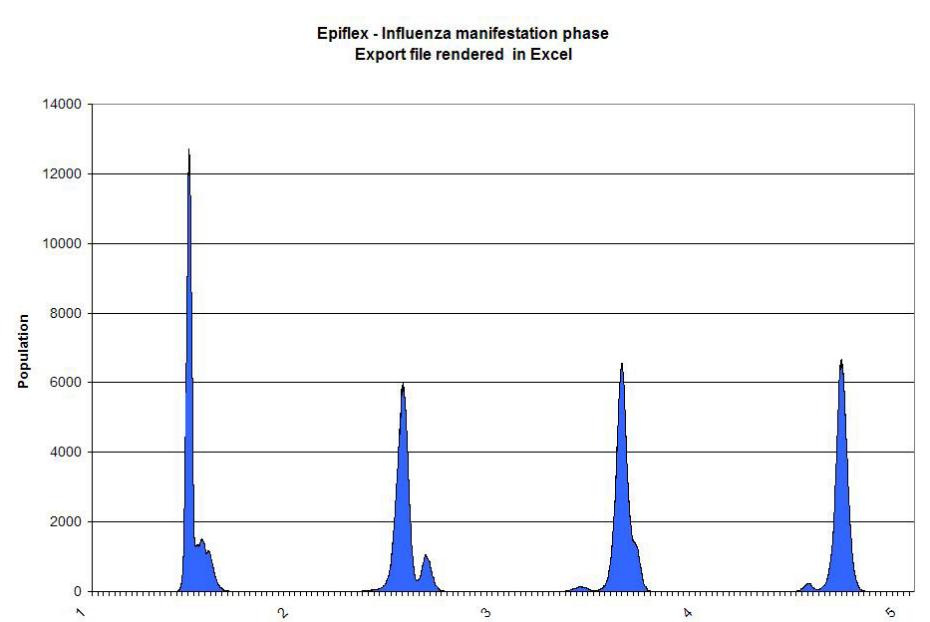


Comparing EpiFlex with surveillance data, we see that WHO/NREVSS surveillance data [36] have a qualitatively similar graph form to EpiFlex for influenza, as shown in Figures 4 and 5.

The width of the primary curves per season for EpiFlex is 3.5 to 4.5 months while that of the NREVSS data is approximately 5 to 7 months, which can be explained by the NREVSS data being collected nationally from surveillance centers, whereas the EpiFlex data shown are for a single area. EpiFlex runs executed with multiple cities connected by transport, such as the 3.5 million population 35 city model, have a combined graph for all cities showing self-similarity to the graphs for individual areas, becoming wider, matching the NREVSS data graph formation.

The NREVSS data consist of diagnostics of samples sent in by physicians. Comparisons of absolute numbers in terms of quantity are therefore not applicable. A percentage of population comparison is done below.

#### Comparison of percentage infected with California surveillance data and other seroprevalence

In Table 1, EpiFlex indicates that roughly 48% of the population has been infected before herd immunity stops the epidemic, though this depends on population size. Total morbidity is obtainable from EpiFlex by adding maximum immune level to deaths, although deaths contribute such a small amount to influenza morbidity that for practical purposes the immune level is used as a proxy for morbidity. Moreover, true morbidity itself is relatively prone to inaccuracy, whereas better measures of immune fractions for influenza are available. The California state average for 2000–2003 is 25.4% infected in a range from 12.7 to 44.6 depending on county [37]. Thus, EpiFlex is above the high end of the state of California estimated morbidity range.

Dowdle [32] gives serological influenza data categorized by age. For influenza A/Swine/15/30 H1, seroprevalence ranges from roughly 25% to over 95%. For influenza A/Hong Kong/68 H3, the range is from 5% to 99%. EpiFlex figures fall within this latter pair of ranges, and EpiFlex immune fraction is more properly comparable.

#### Comparison with SIRP classical modeling

What is most notable in Figure 7 is the relationship between the rough sine wave form of the classically derived SIRP [19] mathematical model and real world Milwaukee data. Contrast this with the graph from the EpiFlex simulator. The SIRP classical type model results are on the left and the EpiFlex simulations are in the right hand chart of Figure 7. Comparing the two, it is clear that once the initial startup period is over for influenza, a repeating wave develops that is similar in overall shape and variability to real world data such as those for Milwaukee, at a roughly similar scale. These two graphs refer to populations that differ in size by about 1 order of magnitude (i.e. Milwaukee is 9 times the size of the model run shown). We can also see a similar number of peaks. Owing to the need to compare these two graphs natively, these two figures are not optimum. However, they show essential features.

**Figure 6:**
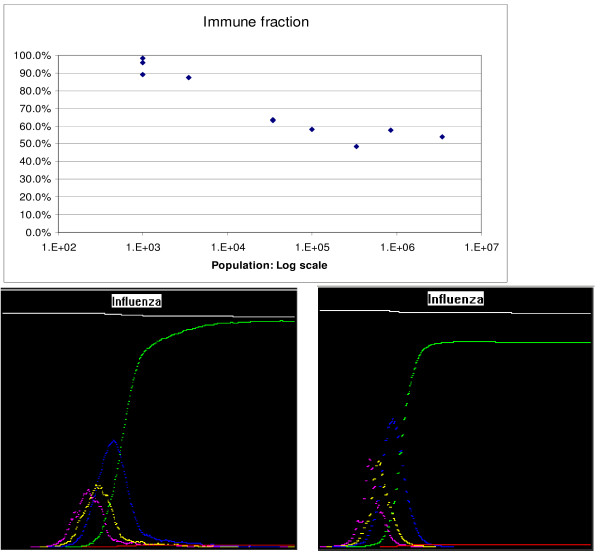
Upper graph shows graphed points for population versus total morbidity as estimated from immunity. Lower two graphs show sample graphs for 3500 (lower left) and 35000 (lower right). The green line in the lower two graphs shows immunity. One vertical demarcation on the x axis is one month in the lower two graphs.

### Example observations

#### Total morbidity rate linked to population size

The smaller a population over the range 1,000 to ~3.5 million, the higher the total morbidity rate, given identical organisms (Figure 6). It is intuitively expected that population size will affect morbidity since, for any given network of contacts connecting individuals in populations, the chance of the epidemic spreading during the window prior to the development of immunity in parts of the population increases as the population size decreases. This effect is most striking when very small populations in the order of 1,000 are examined. Literature data regarding this in real world populations are sparse. However, there are indications from historical accounts of small populations in the new world that a link between population size and morbidity is observable in real world populations [38-43]. The most recent such account is from Heyerdahl in the Pacific in the mid 20^th ^century [44].

**Figure 7:**
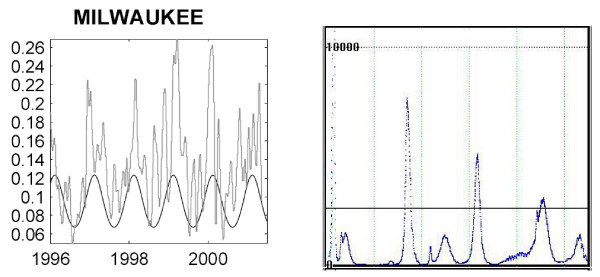
(a. left b. right). Black repeating curve = Hyman-LaForce model Blue = EpiFlex manifestation (morbidity) Light jagged = CDC data from Hyman-LaForce Horizontal black = 2,600 Numeric scale = thousands Horizontal scale = 365 days per demarcation. Time scales are for equal periods of years. (7a.) Results from the SIRP mathematical model of influenza in Milwaukee which is superimposed over CDC data from that paper. The SIRP model of Hyman Laforce was used because its SIRP model for influenza was extended for developing EpiFlex. (7b.) Results from EpiFlex.

In the graphs of Figure 6, the immune fraction at completion is used as a proxy for total morbidity on a log scale of population. The longer an epidemic takes to progress within an enclosed population, the greater the number of potentially infectious contacts that hit a dead end because the host is already immune. Since very small populations will mostly function within the window when there is no host immunity, the infection will spread to a larger fraction. This effect has public health implications because, clearly, the structure of the network is highly significant in determining the likelihood that an infected host will contact naïve hosts. Essentially what this EpiFlex result indicates is that during the period prior to the development of an immune subpopulation, a disease has a functionally higher R_0_. (i.e. R_0 _is variable through the course of an epidemic.)

In the Figure 6 graphs, EpiFlex is also suggesting that there are more asymptomatic infected spreaders of influenza in our populations than surveillance data estimate. This is also suggested by the discussion above regarding comparison with seroprevalence.

#### Difference in peak morbidity related to number of attempted seed events

**Figure 8:**
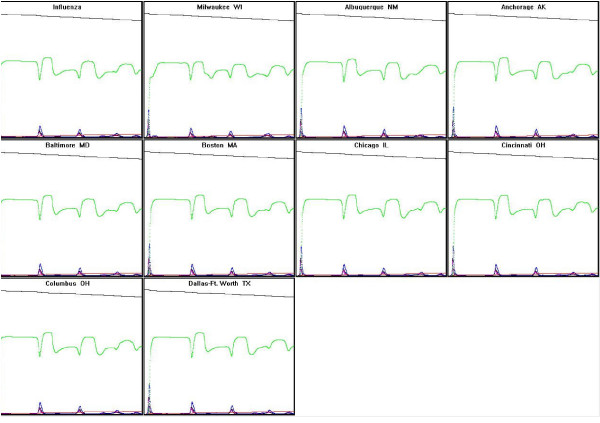
A 5.5 year, 9 city simulation linked by transport corridors. 100,000 people per city.

A minor experimental result is that for a repeating illness such as influenza, when a continuously active initiating disease vector tries to infect 3 people per day, it will develop higher peaks after the initial event than a vector that tries to infect 30 people a day (where both are randomly distributed through the population.) This makes intuitive sense, because there is lower probability that a subpopulation of susceptible hosts will become large when there are more attempts to infect them. Similarly, in a system of cities interconnected by transport linkages, later peaks tend to be smaller and more variable than earlier ones. This is due to two things. First, a degree of low-grade infection linking back through the system provides a higher total level of infection events in the whole system than the formally defined initiating disease vector. Second, as time passes, the mix of immune versus susceptible becomes unsynchronized for the population as a whole, since hosts that escaped infection during one epidemic may be infected during the next, and some whose immunity has declined may also become infected. Thus, it is expected that we would see the development of a complex non-repeating waveform with some similarity. This type of waveform is what EpiFlex shows with longer simulations in large populations, as illustrated in Figure 8.

#### Variety of results for index cases

A variety of results is obtained when one or just a few index cases are provided to seed a single city's susceptible population when those index cases are not repeated. This is expected, owing to random interactions that break the chain of contacts in some percentage of cases. This effect would be expected to increase with a higher skew on super-spreaders. The significance of this for modeled epidemics, particularly in the light of recent work [17], is that in some cases (the proportion would be expected to vary in rough accordance with R_0_) the infection dies out owing to random chance. Thus, a Monte-Carlo model such as EpiFlex, when used in multiple trials, clearly reveals the potential range of variation in epidemics given apparently identical conditions.

#### Wave propagation between cities – manifestation of epidemic

**Figure 9:**
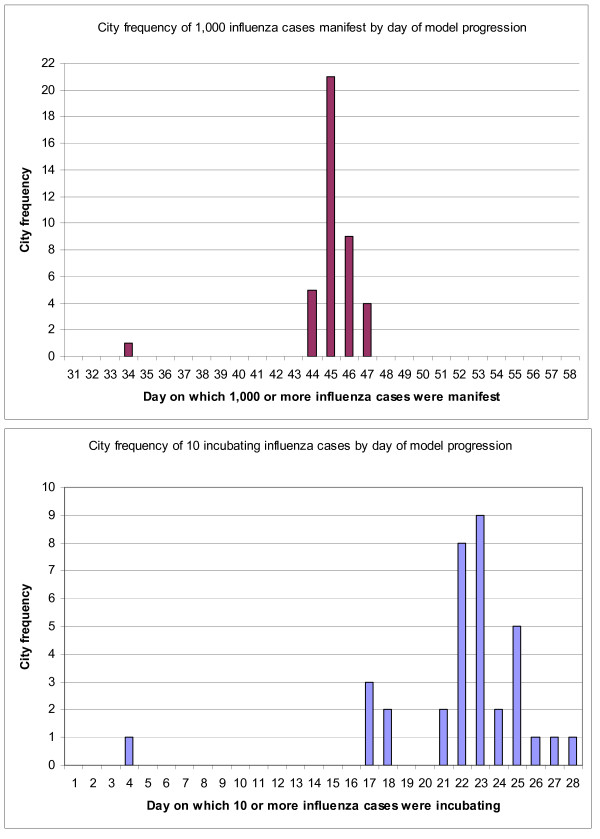
Comparison of distribution of days when 1,000 manifesting cases or more first appear in a city (upper graph – 9a) versus distribution of days when 10 or more incubating cases first appear in a city (lower graph – 9b).

EpiFlex shows wave propagation of epidemics through its transport network that are similar to real world epidemic studies such as those of Viboud et al. [45]. Viboud et al. state a mean duration of 5.2 weeks to spread across the United States, with a range from 2.7 to 8.4 weeks. The EpiFlex results shown using a simplified city configuration of 35 major airline hub cities, with a total 3.5 million population among all 35 cities, shows a propagation wave of 1.8 weeks. While this is shorter than real world data, several factors account for the difference. First, in the current EpiFlex "vanilla" configuration, the transport network is flat in terms of the numbers of persons moved from city to city. Second, each city contains the same population of only 100,000 due to practical limitations. For the propagation histogram of Figure 9a, 1000 manifesting cases or more was used as the data point. Please note, however, this flatness of transport and population is purely a matter of the configuration of the specific model used. The EpiFlex system allows separate specification of all parameters for each city, and any kind of transport level between any two cities that is desired.

#### Wave propagation between cities – incubation versus manifestation of epidemic

Figure 9a shows a histogram of cities in which 1,000 manifesting cases are first occurring. Figure 9b shows a histogram of cities in which the first occurrences of at least 10 incubating cases of influenza appear. Note that in Figure 9b, the duration of spread is 3.4 weeks as opposed to 1.8 weeks for 9a. This ratio of approximately 2X (i.e. 3.4/1.8) is quite possibly significant for real world epidemics. EpiFlex is indicating that during the ramp-up to an epidemic, a significant amount of synchronization occurs between cities.

## Conclusion

EpiFlex is useful for doing in-silico experiments with epidemic behavior and easy to configure. It can run on an ordinary computer without special configuration. Data can be imported from it into other tools and worked with there. It is effective for showing factors that are invisible or difficult to access under normal conditions, such as visibility of incubation and prodromal stages, true morbidity and estimates of immunity. EpiFlex has capabilities that were not discussed owing to time and space constraints. This system is effective in duplicating the kind of multimodality and apparent stochastic variation [13] that are seen in real populations. EpiFlex results can provoke thought about the nature of epidemics and infectious disease spread in interesting ways by providing an experimental test environment that is not as abstracted from its subject as most mathematical models are.

## Glossary

Items in **bold **below are entities that are objects in the EpiFlex system. They are the "nouns", each of which became a class when the program was written in C++. Items in *italics *are actions, the "verbs" that represent interactions between objects.

**Diseases ***infect ***hosts**

**Hosts ***go through the infectious stages*, are members of **Demographic groups **and *move *between **locations**

**Locations ***contain ***hosts **(For example a hospital, home or workplace.)

**Areas ***contain ***locations **(A city, typically) and *contain ***special locations that can move hosts to locations in other areas**.

**Disease Vectors ***introduce ***disease **into **host **populations at some **location **in some **area**, in either a limited way or on a regular cycle.

**Epidemic responses ***modify the spread of an infection*. They represent actions taken to respond to the epidemic that will damp the spread.

## SIRP

**Figure 10:**
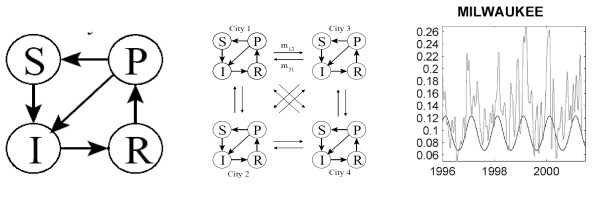
(from left) SIRP Diagram, Multi-city SIRP, Actual versus model.

SIRP is a simple flow chart, which is then elaborated into a multi-city system. Three graphics are reproduced in Figure 10 concluding with output of the model, because the SIRP paper is in a specialty publication and the work was important to the conception of EpiFlex.

As seen in the "SIRP diagram" of Figure 10, a host begins as *susceptible (S) *in the upper left. They can become *infected (I)*, and then they either *recover (R) *or are removed (removal not shown). After *recovery (R)*, they have an immunity level that starts at or near complete as in SIR. This immunity declines, in the case of influenza, over a period of roughly 2 years, with a steep drop-off beginning after the first year, and hence is termed *partial immunity(P)*. (It is not actually the case that the immune system's response to influenza antigens degrades so rapidly; rather, the virus mutates rapidly, and host immunity declines. The net result is similar.) This decline in immunity models the real world antigenic mutation of influenza viruses. In a more perfect model, the influenza virus would change and multiple viruses would be modeled. At some point the immunity declines so that it is effectively zero in the model (which corresponds to large antigenic change) and the person is returned to the fully *susceptible(S) *population. A person can become reinfected (diagonal arrow), though their probability of reinfection is lower, during this period of declining immunity.

In the "Multi-city SIRP" flows of Figure 10 center, *m*_*[x,y] *_denote movement between the cities in a four city example. Moving further to the right of Figure 10 and looking at the "Actual versus model graph", one can see how a sinusoid curve, with some general correlation with period, results from the conventional model.

## Appendix

### Files

There are four files used or generated by EpiFlex. All three generated files have the name of the configuration from the configuration panel appended to the name of the model. After the configuration name is appended the Greenwich Mean Time (GMT) date and time ending with the second.

#### Model file

Model files end in .EPDM. These files are an XML format. This is the file edited by Epiflex using the various panels available from the menu. An EPDM file can also be edited directly in WordPad or a good XML editor by more sophisticated users. Reading through them should be self-explanatory to most users who familiarize themselves with the software.

#### Run record files

Run record files end in .RPX. These files are in text format, comma, and semicolon delimited. This format is intended to be as easy to import as possible. RPX files can be imported into Excel, read by SAS, SPSS, etcetera. At the top of this file is a descriptor of the fields. The most common problem is to import using spaces as a delimiter and have spaces in area names define new columns.

#### Log files

Log files end in .LOG. Everything that happens in a model is logged. These files can get fairly large. Potentially, they could be parsed by a secondary piece of software to match them up with RPX files. However, that is an exercise for the user at this time. There is a viewer for log files under the View menu.

#### Snapshot files

Snapshot files end in .SNAP. Whenever a run completes, a SNAP file is written that is read only. This file is a duplicate of the model that was used to run – as that model existed in memory at completion of the run. This is done as a "lab notebook" aid because it was recognized early on that remembering exactly what was contained in a model was well nigh impossible and a lot of work to document. Note that if your model does not complete its run, the RPX file will still be there, but the SNAP file will not be. In that case it is up to the experimenter to make a record of the state of the model.

The SNAP file can be copied outside of Epiflex, manually changed from read-only and the extension renamed to .EPDM. The resulting model can then be used and run just like any other.

### EpiFlex component developments

Software engineering details such as internal class definitions and structure of components will not be presented since the UI does a better job of educating, and is much more compact.

#### Disease component

**Figure 11:**
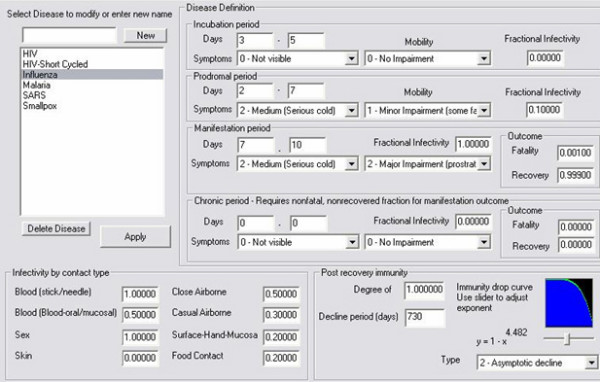
Disease definition panel.

Looking at screen shots of the current UI in Figure 11, one can see what the functional parametric elements of a disease specification are. This particular example is selected for influenza. The parameters set reproduce the known characteristics of the disease.

The disease stages of Figure 11 correspond to the model diagram shown in Figure 1. One can see that this definition is superior in several ways to what is available with SIRP modeling as shown in the previous section. (See Figure 10). In addition to multiple stages, one can define the level of infectiousness for each type of contact that an infected host might have. On the lower right, the partial immunity stage is specified. Here, one is able to define a wide variety of immune responses. Asymptotic decline seems to fit available data and theory on immune system function the best for many diseases. Use of the slider allows one to "eyeball" the asymptotic curve to approximate what seems reasonable. In this case, immunity is set to begin dropping after approximately half the 730 day period has passed. It makes sense to do this because data in this area are fairly sparse, and one may want to perform multiple runs with slightly different immune drop characteristics. Keep in mind that the width of the graph is for the number of days entered on this panel.

#### Host component

A host is implicitly one of the number in the population of an area. In the EpiFlex system, there is one object defining each disease. A host infected by a disease receives a pointer to this archetypal disease object, and stores their disease stages internally as the disease progresses. Most of the functionality of hosts consists of an implementation of disease stage transition.

#### Group component

**Figure 12:**
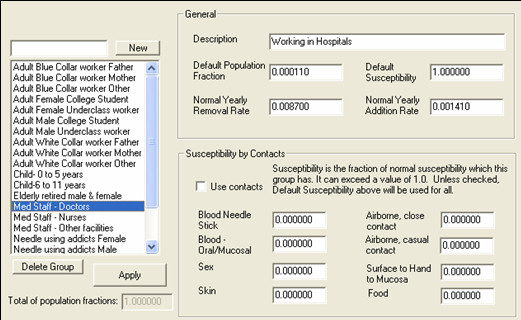
Group demographic population definition panel.

Figure 12 is a screen shot of the UI for a set of demographic definitions selected from US Census data for the nation as a whole. In the resulting model, one can override the default fractions later when using them in populations for specific areas. No fractions are represented that could not be derived from recent census data.

Each demographic group can be set up to specify variations in overall susceptibility to disease, or modify it by specific type of contact if that is desired. In the example, no fractional modifiers have been created for this demographic group. There is no necessity to stick to the demographic groups defined here. One can add new ones as desired.

#### Location component

A location describes a place, the activities that occur there, and the demographic groups that may be drawn there automatically. A location can have a certain number of cells, which are used to specify N identically behaving locations concurrently. This acts as a location repetition count within an area when the location is defined.

A technical point with homes as cells is that the model cannot yet be set to create a number of homes based on population size with a distribution of household sizes. Consequently, one must, at present, define the number of home cells appropriate to hold the population for each area. If a distribution function were available to specify household sizes and households were definable by population size, it would then be practical to provide canned profiles of household sizes based on US census data.

**Figure 13:**
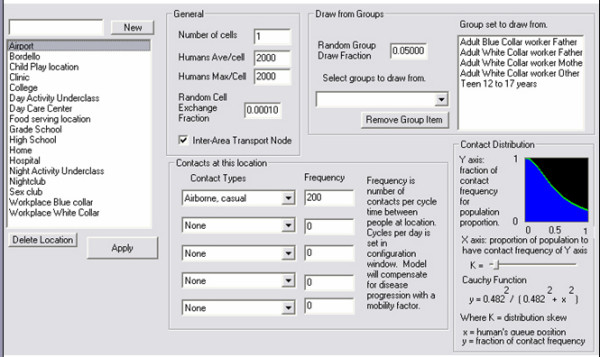
Location definition panel.

The types of contacts that are available among hosts in the location are defined, together with the average number of such contacts per host during one cycle of the model; see Figure 13. To the right of the contact definitions is a skew function that enables one to specify the degree to which contacts vary. Within any host subpopulation, there are high contact individuals and lower contact individuals. The example shown sets the difference rather high to describe the way that service workers interact more strongly than customers. When running the simulation, during determination of which hosts become infected in this cycle, the position of the host in the queue is used together with the contact frequency to decide how many contacts an individual will get in a particular cycle. Think of this adjustable skew graph as the histogram of contact counts.

**Figure 14:**
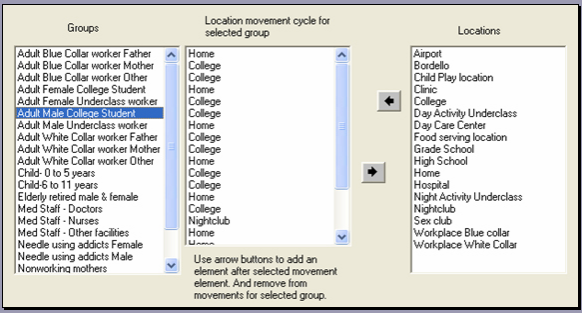
Group movement cycle definition panel.

For each demographic group, a cycle of movement between locations in an area is defined as illustrated in Figure 14. In the model used as an example, days are divided into three cycles. So a sequence of 21 movements defines a 7 day week. Once a cycle completes, it restarts at the top. This is how hosts in the model are moved from one location to the next.

#### Area component

**Figure 15:**
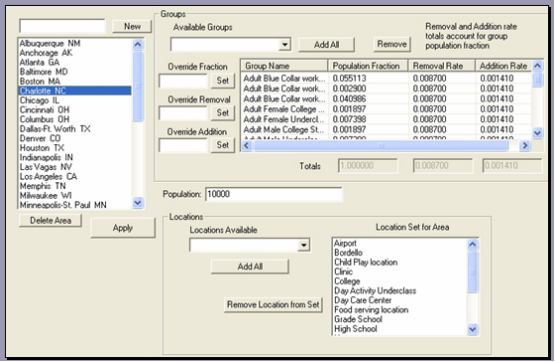
Area definition panel.

An area is made up of a list of locations that it contains, as shown in Figure 15. An area specifies a particular population level, and the population fractions for each demographic group making up the area are allocated at runtime. Demographic population, removals and additions can be overridden at the area level.

**Figure 16:**
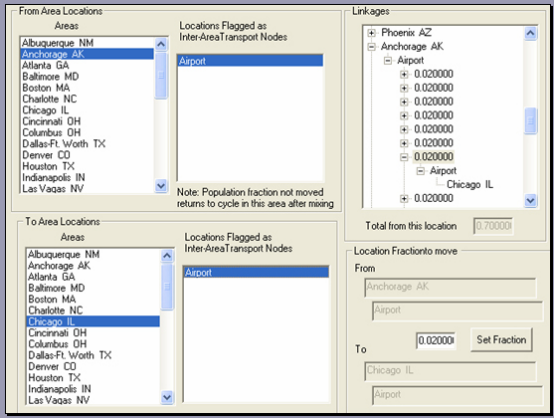
Area linkages panel.

An area also has linkages to other areas, through special locations that are eligible as links. In the example of Figure 16, the Anchorage airport is going to send 2% of its population to Chicago, Illinois, each cycle. In each cycle, 70% of the population of the Anchorage airport location is going to be shipped off somewhere.

#### Initiating disease vector component

**Figure 17:**
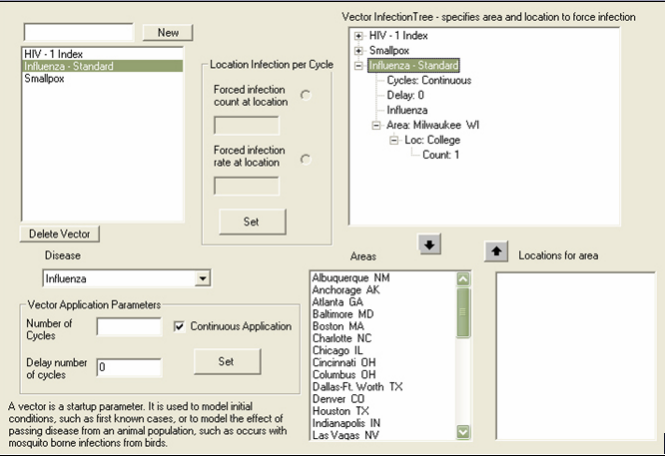
Initiating disease vector definition panel.

A 'Vector' is defined in EpiFlex as *n *infection events to attempt at a specific location. A vector can repeat each cycle, or have a limited input. A vector can represent an arthropod or snail, or it can represent people arriving on aircraft from Hong Kong. The vector shown in Figure 17 is a startup vector for influenza used for these modeling runs. This initiating disease vector will operate for the first 3 cycles, then stop, attempting to produce 9 cases at a location in Chicago. An initiation disease vector can have delay parameters, run continuously, or run once. It can force the infection of a specific number of people or infect them at a particular rate.

#### Response component

When a disease occurs, the medical system will respond to it in some way. Some illnesses, such as influenza A, will result in very little intervention. Others, such as SARS or smallpox, would result in a great deal of intervention very rapidly. Consequently, a reasonable modeling system will allow response to an epidemic occurrence to be modeled.

**Figure 18:**
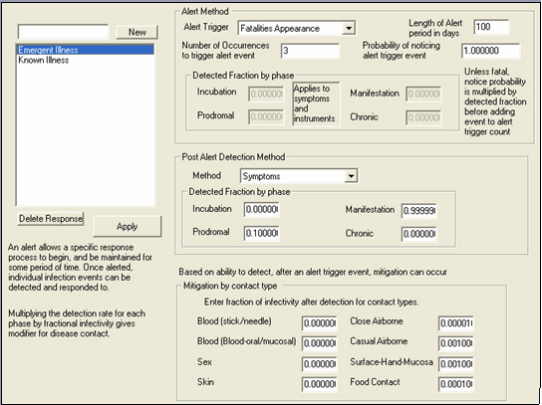
Epidemic response definition panel.

In the example shown in Figure 18, we see the response specification for an emergent illness. A response has an alert trigger, which in this case is the appearance of fatalities. This results in a period of heightened awareness of the disease and its symptoms, which is set in this example to 100 days. The probability of noticing the triggering event is set, to unity in this example, which may be overly optimistic. This specification says that after three fatalities, an alert would be triggered. Since this alert trigger is for fatalities, not symptoms or instruments, the detection fraction for each disease stage is not applicable.

Once the alert has been triggered, the detection method for the illness switches primarily to symptoms. We can then specify what fraction of each occurrence will be diagnosed and at what stage. If we detect an event, then we can mitigate it. In this implementation, one has to specify the degree of infectiousness remaining after intervention is in process. Some methods, for some diseases, can stop disease spread completely. This is not true for all diseases, so it must be specifiable.

Note that this component is the one with which the author is least comfortable. None of the examples for this paper have used responses as a consequence. The primary reason is that the way response is modeled is over-simplified and does not conform well to the response that happens in the real world; see: Limitations of EpiFlex modeling in the introduction.

### Walkthrough

#### Overview

EpiFlex is now composed of 83 major classes, of which 45 are core internal model functionality, the rest being infrastructure and UI. A functional walkthrough of how the system operates is presented below.

#### Verification

A model starts running by verifying the model data. It looks for any references to things that were deleted and definitions that are impossible. It writes an error log file with this information that can be audited after the run. If errors are found, you will be informed of their severity and given the option to cancel. Many of these errors are non-fatal, and may be modified with a warning, but they may change the results of a run.

#### Initialization

The EpiFlex system iterates through all areas in a model and allocates hosts, putting them in their initial locations, per the movement definitions for the demographic group.

Each group steps through its location movement list to determine whether the area to which it is attached has the locations the group needs. If it does not, EpiFlex will increment the pointer when it comes to this location, but it will leave the hosts in their previous real location until a good one is found. This was done for ease of practical use, allowing the same demographic to be used when a location may not exist in an area.

#### Cycle

Step 1 – disease stage pass. For each infected host, it will update the stage of the illness for that host. This is also where the system checks to see if disease response conditions have been met yet.

Step 2 – vector pass. EpiFlex will iterate through each vector and apply the disease to the locations in each area in the vector according to the rules defined.

Step 3 – infection pass. Iterate through each location in each area, and figure out the contacts between infected and uninfected hosts. For each contact between an infected host and a host not infected with the disease, a probabilistic determination will decide whether or not this illness is communicated.

Step 4 – iterate areas looking for locations that have group draw factors. Randomly pull hosts from the groups specified, and move them to the group draw location. This feature is used to simulate various things; generally an airport will have this type of specification.

Step 5 – iterate all areas looking at area links. Area links push population from one location to another area's location as specified.

Step 6 – Areas will be iterated, and each location in each area will be iterated using the random exchange factor to move hosts assigned to cells of a location between cells in that location.

Step 7 – Normal addition and removal of population groups is applied to model. This allows the user to model normal birth and death rate plus immigration and emigration across the outer boundary of the model.

Step 8 – iterate all areas, and iterate through each location in each area. For each location, it will iterate each host attached to the location. Each host has a location pointer for its group that indicates where it is in its movement cycle. It increments the pointer, and if the location name is different from the one it is in, it will find a location of that name in the area to put itself into. If the location has N cells, it will put itself into the cells in a semi-randomized fashion according to the parameters defined. Note that currently the hosts do not remember which cell they are put into from one iteration to another. So they will not return to the same cell next time they come back around in their movement cycle. This is a limitation at present, which would require greatly increased space allocations to change.

Step 9 – An audit is done to verify that all hosts occur only once in a location.

Step 10 – Areas are iterated, locations are iterated, to total results. Results are then either written to the log file and/or displayed on the main window in a graph form depending on how parameters were specified.

Final – When a model run completes, the .RPX data file is closed, a .SNAP snapshot of the model is made as a read only file to record exactly what parameters gave rise to the data, and the log file is closed.

## Supplementary Material

Additional File 1 containing software and models. Use of Epiflex software for research publications requires citation to this paper. Click here for file
